# South Asians Have Elevated Postexercise Blood Pressure and Myocardial Oxygen Consumption Compared to Europeans Despite Equivalent Resting Pressure

**DOI:** 10.1161/JAHA.111.000281

**Published:** 2012-10-25

**Authors:** Nish Chaturvedi, Rajaram Bathula, Angela C. Shore, Ronney Panerai, John Potter, Jaspal Kooner, John Chambers, Alun D. Hughes

**Affiliations:** National Heart and Lung Institute, Imperial College Academic Health Sciences Centre, London, UK (N.C., R.B., J.K., J.C., A.D.H.); Institute of Biomedical and Clinical Science, Peninsula Medical School, Exeter, UK (A.C.S.); Department of Cardiovascular Sciences, University of Leicester, UK (R.P.); School of Medicine Health Policy and Practice, University of East Anglia, UK (J.P.)

**Keywords:** autonomic function, blood pressure, epidemiology, glucose, pulse wave velocity

## Abstract

**Background:**

Stroke mortality rate is higher in South Asians than in Europeans, despite equivalent or lower resting blood pressure (BP). Elevated recovery BP after exercise predicts stroke, independently of resting values. We hypothesized that South Asians would have adverse postexercise hemodynamics and sought explanations for this.

**Methods and Results:**

A population-based sample of 147 European and 145 South Asian middle-aged men and women performed the Dundee 3-minute step test. Cardiovascular risk factors were measured. BP, heart rate, and rate–pressure product, a measure of myocardial oxygen consumption, were compared. With 90% power and 5% significance, we could detect a difference of 0.38 of a standard deviation in any outcome measure. Resting systolic BP was similar in South Asians (144 mm Hg) and Europeans (142 mm Hg) (*P*=0.2), as was exercise BP (*P*=0.4). However, recovery systolic BP at 3 minutes after exercise was higher in South Asians by 4.3 mm Hg (95% confidence interval [CI], 0.2 to 8.3 mm Hg; *P*=0.04). This effect persisted when adjusted for exercise BP and work effort (5.4 mm Hg [95% CI, 2.2 to 8.7 mm Hg; *P*=0.001]). Adjustment for baroreflex insensitivity and greater aortic stiffness in South Asians contributes greatly to attenuating this ethnic difference (1.9 mm Hg [95% CI, −0.9 to 4.6 mm Hg; *P*=0.4]). Similarly, rate–pressure product recovery after exercise was impaired in South Asians by 735 mm Hg/min (95% CI, 137 to 1334 mm Hg/min; *P*=0.02); again, adjustment for baroreflex insensitivity and aortic stiffness attenuated this difference (261 mm Hg/min [95% CI, −39 to 561 mm Hg/min; *P*=0.3]).

**Conclusion:**

Postexercise recovery of BP and rate–pressure product is impaired in South Asians compared to Europeans even though resting and exercise BP are similar. This is associated with the autonomic dysfunction and aortic stiffness in South Asians. **(*J Am Heart Assoc*. 2012;1:e000281 doi: 10.1161/JAHA.111.000281.)**

## Introduction

People of South Asian descent are particularly prone to stroke. Rates of death from stroke for people of South Asian descent in the United Kingdom are between 20% and 250% greater than those of the general population.^1,2^ High blood pressure (BP) is the dominant risk factor for stroke within populations, with a population-attributable risk between 50% and 73%.^3,4^ Nevertheless, between-population comparisons show that resting BP in all South Asian subgroups is equal to or lower than resting BP in Europeans.^5^

However, BP change on exercise and recovery might be more representative of daily physical stresses and has additional prognostic value over resting BP. Typically, BP rises during exercise and falls in recovery, sometimes to levels below resting. The utility of maximum BP or change in BP on exercise in predicting cardiovascular disease (CVD) is controversial^6–10^; a large study of asymptomatic individuals concluded that peak exercise BP was not predictive independently of resting BP.^11^ More consistently, impaired BP recovery after exercise predicts adverse outcomes^12–14^ more strongly than exercise or resting BP.^15^ Heart rate also changes on exercise,^16^ and parallel changes in BP and heart rate, calculated as the rate–pressure product (RPP), provide an indirect measure of myocardial oxygen uptake, reflecting the adequacy of cardiac response to metabolic demand.^17^ Both attenuated RPP responses to exercise^18^ and poor postexercise recovery RPP^19^ predict CVD.

We hypothesized that, compared to Europeans, South Asians would have impaired BP recovery after exercise, not explained by resting BP. A secondary objective of our study was to determine whether RPP recovery after exercise was attenuated in South Asians. Finally, we explored the role of cardiovascular risk factors in explaining ethnic differences.

## Methods

### Study Design

Because ethnic differences in stroke are observed at a general population level, explanations must be sought in an equivalently representative population sample. Restriction to an extreme high-risk sample or, alternatively, exclusion of all individuals with morbidity or abnormal risk factors and individuals on medication would result in unrepresentative, biased findings that would not be generalizable. We recruited from the London Life Science Prospective Population Study (LOLIPOP). Details of LOLIPOP have been published.^20^ In brief, >30 000 men and women 35 to 75 years of age were recruited from 58 primary care registers in West London to provide a community-based sample. Because primary care is the gateway to health care and registration is free, this forms the most representative sampling frame for health research in the United Kingdom. Participants were invited to come to a local clinic for cardiovascular assessment and to provide a blood sample for DNA. Ethnicity (South Asian or European) was assigned by country of birth of all 4 grandparents. The response rate was 62%, and participants consented to be recontacted for future studies. The LOLIPOP atherosclerosis cohort substudy aimed to recruit ≈2000 individuals who were free of clinical CVD for more detailed investigation. LOLIPOP participants were invited when they attended their initial clinic visit, and a total of 2293 individuals completed this substudy, which included ultrasound for intima-media thickness and echocardiography.^21^ These latter individuals formed our sampling frame. Because resting BP differs by South Asian ethnic subgroup,^22^ we restricted our sample to people of Punjabi Sikh origin, as this group forms 60% of the South Asian population in this part of West London. Additionally, women <55 years of age (ie, likely to be premenopausal) were excluded. Our sampling was then stratified by ethnicity, sex, and 5-year age group. Because premenopausal women were excluded, we designed our study to be two thirds male. We required 300 participants, and mailshots (bulk mailouts to selected participants) were sent out in batches until the required sample was achieved. A total of 746 letters of invitation were sent, and 356 (48%) agreed to participate. There were no marked differences in health characteristics between responders and nonresponders: Mean age was 62.0±6.7 and 61.2±7.8 years (*P*=0.1); systolic/diastolic pressures, 134±19.4/80±9.9 and 136±19.6/80±10.3 mm Hg (*P*=0.2/0.4); fasting glucose, 5.8±2.0 and 5.8±2.0 mmol/L (*P*=0.9); HbA_1c_, 6.0±1.2% and 6.0±1.1% (*P*=0.9); and hypertension prevalence, 38% and 36% (*P*=0.6), respectively. Exclusion criteria for our study were clinical CVD, atrial fibrillation, valve disease, hormone replacement therapy, and carotid artery stenosis, because these interfere with study assessments. Of the 356 respondents, 11 were excluded because of hormone replacement therapy use, 8 because of atrial fibrillation, and 5 because of stroke. Thirty-two respondents replied too late to be included in our study sample. The study was approved by the local ethics committee, and all participants gave written informed consent.

### Study Investigations

Participants attended the clinic after an overnight fast. Ethnicity was confirmed with the participant and was based on self-assessment and place of birth of all grandparents. A questionnaire included items on types and durations of activity at home and work throughout the year to generate a physical activity score. Height, weight, and waist and hip circumferences were measured, and sitting brachial BP was measured 3 times with the use of a validated automated device (OMRON 705CP) after >5 minutes rest.^23^ The average of the final 2 readings was used in analysis. An exercise test was performed with the Dundee step test.^24^ This involved stepping up, starting with the dominant foot, to a step 17.5 cm high. The nondominant foot then stepped up, before each foot stepped down in turn. A metronome was used to ensure that participants performed the exercise to a similar intensity by completing a step cycle every 2 seconds. The step test was continued for 3 minutes. A single standing BP and heart rate measurement was made before exercise commenced and was repeated while the participant was still standing at the end of exercise. The participant then was instructed to lie down, and a further measurement was performed after 3 minutes of recovery. Work (W; given in kilograms per meter per minute) performed during the step exercise was calculated as follows^25^:


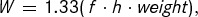


where *f*=steps/minute, *h*=height of step in meters, and *weight* is given in kilograms. The exercise test could not be performed by 2 Europeans and 6 South Asians because of mobility problems; their baseline characteristics did not differ from the rest.

After 30 minutes of rest in a temperature-controlled room, autonomic function tests were performed^26^ to measure baroreflex sensitivity, which is of particular relevance to BP responses to stressors.^27^ Sitting central BP was measured by applanation tonometry with a Sphygomcor device (Atcor Medical, West Ryde, Australia) at the radial artery. Pulse wave velocity (PWV) was measured between the carotid and femoral sites by using an ECG-gated ultrasound “foot-to-foot” method (Pulse Trace PT 2000, Micromedical Ltd, Kent UK).

Fasting blood samples were analyzed for glucose (Hexokinase method; Roche, Basel, Switzerland), HbA_1c_ (nonenzymatic method; Toshoh, Tokyo, Japan), lipids (enzymatic colorimetric method; Roche, Basel, Switzerland), and insulin (enzyme-linked immunosorbent assay; Roche, Indianapolis, IN). Homeostatic model assessment (HOMA) indices of insulin resistance^28^ were calculated.

We wished to power our study for an ethnic difference in recovery BP or RPP that would account for the 50% greater stroke mortality rate in South Asians, but there are few studies that quantify the association between exercise-related hemodynamic parameters and stroke outcomes. Previously, a standard deviation of 1.0 in exercise BP parameters has predicted an increased risk of stroke over ≈10 years of follow-up ranging from 1.38- to 2.33-fold, depending on whether exercise or recovery BP was the determinant, and depending on the duration of exercise and duration of recovery.^15^ This range of excess risk of stroke in association with a given increment in exercise-related BP is similar to the observed ethnic difference in mortality rate and therefore was the magnitude of difference we would wish to seek. However, individuals recruited to that previous study^15^ were younger than those recruited in the present study, and the exercise stressor was a bicycle test rather than the Dundee step test. Therefore, to ensure that we did not miss a clinically important difference, and recognizing the differences between our own study and previous work, we opted for a sample size that would enable a more conservative difference in exercise hemodynamic parameters to be detected. With 150 individuals in each ethnic group, we were powered to detect a difference of at least 0.38 of a standard deviation in any BP or RPP outcome measure, with 90% power allowing for some data loss. STATA version 11.1 (StataCorp, College Station, TX) was used for analysis. Baseline characteristics are presented as means±standard deviations, or as medians (25th, 75th percentiles) for skewed data and percentages for categorical data. For correlation and multivariable analysis, skewed data were log-transformed. Pearson correlation coefficients were calculated to demonstrate associations between key variables and our outcomes of recovery systolic BP and RPP. We first forced variables into a core model that took account of a priori confounders. These included age, sex, work effort, and either systolic BP or RPP at the end of exercise, as appropriate. Variables that were hypothesized a priori to account mechanistically for the ethnic difference in recovery parameters then were added individually to this core model, and their effect on attenuating the ethnic difference was determined. Correlated variables (eg, measures of glycemia and insulin resistance) were individually tested, and the single variable that provided the greatest attenuation of the ethnic difference was chosen for presentation. A final multivariable model was built that added all variables that best attenuated (ie, accounted for) the ethnic difference, and likelihood ratio testing was used to compare model fit.

## Results

Mean age was ≈62 years (Table 1). Sitting systolic pressure was just 2 mm Hg greater in South Asians than in Europeans, and diastolic BP, at 82 mm Hg, was identical in the 2 groups. Lying and standing brachial BP and central systolic BP were also similar in both ethnic groups (Figure, 1A), although South Asians reported more antihypertensive medication use. This latter was due to the greater diabetes mellitus prevalence in South Asians; antihypertensive medication is instituted at lower BP levels in individuals with diabetes mellitus than in those without diabetes mellitus. South Asians had higher indices of glycemia and insulin resistance, higher heart rates, and lower baroreflex sensitivity. Although not statistically significant, carotid-femoral PWV was higher in South Asians. The work effort performed during the step test was significantly lower in South Asians than in Europeans.

**Table 1.** Demographic and Clinical Characteristics by Ethnicity*

Variable	Europeans (n=147)	South Asians (n=145)	*P*
Age, y	62.6±6.6	62.0±6.3	0.4

Male, n (%)	94 (66)	96 (69)	0.6

PWV, carotid to femoral, m/s	9.3±1.9	9.6±1.9	0.2

Antihypertensive medication, n (%)	36 (24)	56 (39)	0.009

β-Blocker, n (%)	5 (3)	11 (8)	0.1

Diuretic, n (%)	12 (8)	12 (8)	1.0

Renin–angiotensin–aldosterone system blocker, n (%)	22 (15)	39 (27)	0.01

Diabetes, n (%)	11 (8)	39 (27)	<0.0001

Oral hypoglycemic medication, n (%)	7 (5)	32 (22)	<0.0001

Insulin medication, n (%)	3 (2)	5 (3)	0.5

Height, cm	170±9.5	166±9.4	0.0004

Weight, kg	80±16.5	74±12.9	0.0006

Body mass index, kg/m^2^	27.6±5.0	26.9±4.2	0.2

WHR	0.93±0.10	0.97±0.10	0.001

Work effort, kg/m/min	551 (481, 640)	510 (452, 572)	<0.0001

Ever smoker, n (%)	90 (62)	7 (4)	<0.0001

Physical activity, kJ/wk	11.6±4.9	10.3±4.3	0.03

TV watching ≥9 h/wk, n (%)	111 (78)	73 (51)	<0.0001

Secondary school education or less, n (%)	91 (67)	47 (34)	<0.0001

Glucose, mmol/L	5.2±0.9	5.5±1.4	0.02

HbA_1c_, %	5.8±0.90	6.4±1.1	<0.0001

Insulin, pmol/L	5.4 (3.7, 8.8)	7.6 (4.7, 12.0)	0.005

HOMA-estimated insulin resistance	1.18 (0.80, 2.12)	1.79 (1.02, 3.01)	0.003

Triglyceride, mmol/L	1.32 (0.98, 1.92)	1.48 (1.04, 2.04)	0.1

Total cholesterol, mmol/L	5.5±1.1	5.2±1.1	0.01

High-density lipoprotein cholesterol, mmol/L	1.41±0.4	1.31±0.34	0.04

Sitting heart rate, bpm	62±9.7	65±11.3	0.008

Baroreflex sensitivity, ms/mm Hg	6.46 (4.97, 9.24)	5.62 (4.08, 8.52)	0.02

Mean RR interval, ms	1022±148	974±146	0.007

* Values given as mean ± standard deviation for normally distributed data, median (25th, 75th percentiles) for skewed data, and number (percentage) for categorical data.

**Figure 1.**
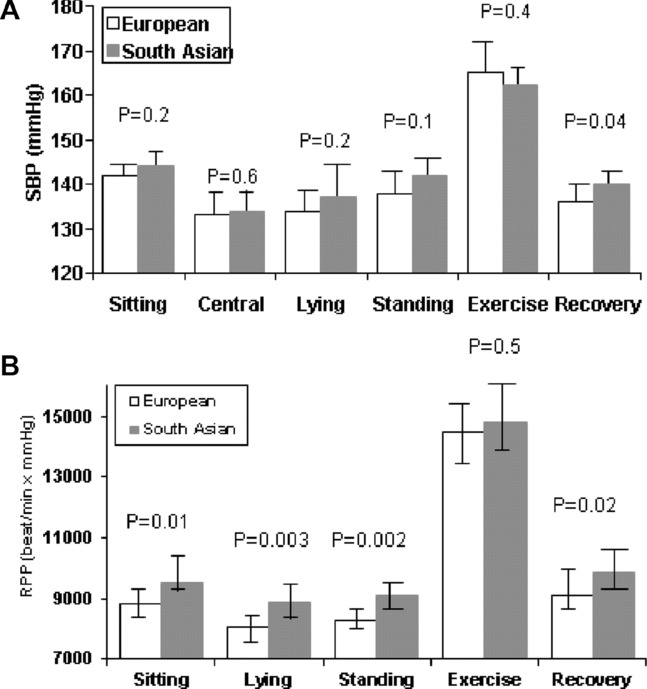
A, Resting, exercise, and recovery systolic BP by ethnicity. SBP indicates systolic BP. B, Resting, exercise, and recovery RPP by ethnicity.

Recovery systolic BP 3 minutes after exercise was 4.0 mm Hg higher in South Asians (95% CI, 0.2 to 8.3 mm Hg) than in Europeans (*P*=0.04; Figure, 1A). Recovery BP was strongly correlated in both ethnic groups with age, work effort, glycemia and insulin resistance, waist–hip ratio (WHR), other measures of BP, PWV, and autonomic function (Table 2). Recovery systolic BP remained significantly higher in South Asians (5.4 mm Hg [95% CI, 2.2 to 8.7 mm Hg; *P*=0.001]) when adjusted for key confounders in the core model (Table 3). Individual risk factors that attenuated the ethnic difference in recovery BP when added singly to the core model included sitting BP, antihypertensive medication, measures of glycemia and insulin resistance (the best being HbA_1c_), baroreflex sensitivity, WHR, and PWV. Habitual physical activity did not contribute. The final multivariable model included baroreflex sensitivity, PWV, sitting BP, and use of antihypertensive medication, all of which made significant and independent contributions. This reduced the ethnic difference in recovery systolic BP to 1.9 mm Hg (95% CI, −0.9 to 4.6 mm Hg), rendering it not statistically significant (*P*=0.4). The effects of both HbA_1c_ and WHR, although strong and significant individually, were overwhelmed by the other variables in the model, specifically PWV and baroreflex sensitivity. HbA_1c_ and WHR were no longer themselves statistically significant, and their inclusion did not further attenuate the ethnic difference in recovery BP and did not improve model fit.

**Table 2.** Pearson Correlation Coefficients (*r*) Between Systolic Pressure and RPP on Recovery After Exercise and Cardiovascular Risk Factors by Ethnicity

	Recovery Systolic BP		Recovery RPP	

	European	South Asian	European	South Asian
Variable	*r*	*r*	*r*	*r*
Age	0.21‡	0.30‡	0.13	0.17†

Work effort*	0.38§	0.18†	0.32§	0.21‡

Glucose	0.16†	0.28§	0.25‡	0.29§

HbA_1c_	0.11	0.40§	0.17†	0.33§

Insulin*	0.21‡	0.19†	0.38§	0.29§

HOMA-estimated insulin resistance*	0.22‡	0.25‡	0.39§	0.34§

Height	0.31§	0.13	0.05	0.12

Weight	0.38§	0.18†	0.32§	0.21‡

Body mass index	0.25‡	0.10	0.34§	0.13

WHR	0.39§	0.20‡	0.30§	0.15

Exercise score	0.15	−0.05	−0.08	−0.09

Sitting systolic BP	0.66§	0.78§	0.55§	0.62§

Central systolic BP	0.59§	0.69§	0.43§	0.45§

Sitting heart rate	0.18†	0.29§	0.66§	0.73§

Exercise systolic BP	0.73§	0.56§	0.59§	0.45§

Exercise RPP	0.58§	0.48§	0.81§	0.74§

PWV	0.50§	0.53§	0.51§	0.52§

Baroreflex*	−0.24‡	−0.40§	−0.45§	−0.48§

Mean RR interval	−0.19†	−0.26§	−0.67§	−0.69§

* Log-transformed variables.

† *P*≤0.05

‡ *P*≤0.01

§ *P*≤0.001.

**Table 3.** Multivariable Models of the Impact of Risk Covariates on Ethnic Differences (South Asian–European) in Recovery Systolic BP and Recovery RPP

	Recovery Systolic BP			Recovery RPP		

Model variables	β Coefficient	95% CI	*P*	β Coefficient	95% CI	*P*
Core model						

Ethnicity	5.4	2.2 to 8.7	0.001	645	252 to 1038	0.001

Age	0.5	0.3 to 0.8	0.0001	28	−1.8 to 58.5	0.07

Sex (female–male)	−6.8	−10.4 to 3.2	<0.0001	−151	−589 to 286	0.5

Work effort	0.01	−0.01 to 0.02	0.5	0.7	−1.4 to 2.8	0.5

Exercise systolic BP	0.4	0.3 to 0.5	<0.0001	…	…	…

Exercise RPP	…	…	…	0.44	0.40 to 0.49	<0.0001

Variables added to core model, β coefficients shown for ethnicity and added variables only						

Ethnicity	4.0	1.3 to 6.6	0.003	305	14 to 596	0.04

Sitting systolic BP	0.55	0.46 to 0.64	<0.0001	…	…	…

Sitting RPP	…	…	…	0.66	0.58 to 0.74	<0.0001

Ethnicity	4.2	1.0 to 7.4	0.01	552	157 to 946	0.006

Antihypertensive drugs	6.9	3.5 to 10.3	<0.0001	578	154 to 1002	0.008

Ethnicity	4.0	0.6 to 7.4	0.03	510	98 to 923	0.02

HbA_1c_	2.3	0.7 to 3.9	0.005	215	22 to 408	0.03

Ethnicity	3.7	0.6 to 6.7	0.02	498	123 to 872	0.009

PWV	3.2	2.3 to 4.1	<0.0001	378	264 to 491	<0.0001

Ethnicity	3.2	−0.03 to 6.5	0.052	336	−31 to 704	0.07

Baroreflex	−8.0	−11.1 to −4.8	<0.0001	−1205	−1568 to −842	<0.0001

Ethnicity	2.8	−0.5 to 5.9	0.1	511	107 to 915	0.01

WHR	15	−5 to 34	0.1	1270	−1102 to 3642	0.3

Ethnicity	5.2	1.9 to 8.5	0.02	617	223 to 1010	0.002

Physical activity	0.03	−0.31 to 0.38	0.9	−41	−82 to 0	0.053

Final multivariable model						

Ethnicity	1.9	−0.9 to 4.6	0.4	261	−39 to 561	0.3

Sitting systolic BP	0.4	0.3 to 0.5	<0.0001	…	…	…

Sitting RPP	…	…	…	0.52	0.41 to 0.62	<0.0001

PWV	1.6	0.8 to 2.5	<0.0001	165	68 to 261	0.001

Baroreflex	−3.8	−6.4 to −1.1	0.006	−356	−689 to −24	0.04

Antihypertensive drugs	3.2	0.3 to 6.1	0.03	…	…	…

Statistical significance was retained when analyses were restricted to participants not on antihypertensive medication (5.5 mm Hg [95% CI, 0.8 to 10.2 mm Hg; *P*=0.02]) and when the few people on β-blockers were excluded. Similarly, South Asians retained their greater recovery systolic BP (4.7 mm Hg [95% CI, 0.4 to 8.9 mm Hg; *P*=0.03]) when those not on oral hypoglycemic medication were analyzed separately.

Recovery diastolic BP showed similar trends to systolic BP, being 1.8 mm Hg (95% CI, −0.2 to 3.7 mm Hg) higher in South Asians (*P*=0.08) and increasing to 2.9 mm Hg (95% CI, 1.2 to 4.6 mm Hg; *P*=0.001) after adjustment for exercise diastolic BP and work effort.

Recovery heart rate was higher in South Asians than in Europeans (adjusted for age and sex) by 3.4 bpm (95% CI, 0.4 to 6.4 bpm; *P*=0.03). This ethnic difference was abolished (0 bpm [95% CI, −1.8 to 1.9 bpm; *P*=1.0]) when adjusted for resting heart rate.

RPP was greater in South Asians on sitting, lying, standing, and recovery (Figure, 1B) but did not differ after exercise. Recovery RPP was 735 mm Hg/min (95% CI, 137 to 1334 mm Hg/min) higher in South Asians than in Europeans (Figure, 1B; *P*=0.02). This difference remained statistically significant when adjusted for confounders in the core model (Table 3). Further adjustment for sitting RPP significantly attenuated the ethnic difference in recovery RPP, as did measures of hyperglycemia, baroreflex sensitivity, and PWV (Table 3). Multivariable analysis showed that sitting RPP (*P* < 0.0001), baroreflex sensitivity (*P*=0.04), and PWV (*P*=0.001) each independently contributed to explaining the ethnic difference in recovery RPP.

## Discussion

BP and RPP, a measure of myocardial oxygen uptake, fail to recover effectively after modest exercise in South Asians. This effect remained statistically significant when adjusted for ethnic differences in work done, resting BP, and antihypertensive medication. The ethnic difference in hemodynamic recovery was abolished when adjustment was made for the poorer baroreceptor activity and increased aortic stiffness in South Asians.

Previous studies, generally in high-risk individuals, reported strong associations between blunted BP response to exercise and CVD,^12–14^ with recovery BP appearing to be a better predictor than resting or maximal exercise BP.^15^ True population-based studies are few but suggest a 50% excess cardiovascular risk in association with impaired recovery BP, even when other risk factors, including resting BP, are accounted for.^29^ Previous studies have used different exercise stressors, making it difficult to contextualize the potential impact on stroke risk of the 4–mm Hg elevation in systolic BP after exercise that we find here in South Asians. However, for resting BP, this difference would be expected to increase stroke risk by ≈40%.^30^ Blunted recovery RPP after exercise also predicts CVD, often independently of other risk factors.^18,19,31^

The stimulus for this study was the paradox of elevated stroke mortality rate in South Asians despite similar resting BP compared to Europeans. Because BP determines around three quarters of the population-attributable risk of stroke, higher resting BP in South Asians would be anticipated.^4^ There is a gradient of increasing relative excess stroke mortality rates in South Asian migrants to the United Kingdom from India (1.16%), Pakistan (1.41%), and Bangladesh (2.49%) compared to Europeans.^2^ In contrast, resting systolic BP in population surveys displays a reverse trend to stroke mortality rate, being lowest in Bangladeshi men (127 mm Hg), intermediate in Pakistani (130 mm Hg) and Indian (134 mm Hg) men, and highest in European-origin men (137 mm Hg). Prospective studies suggest that resting systolic BP would need to be ≈5 to 10 mm Hg and diastolic BP ≈2.5 to 5 mm Hg higher in South Asians than in Europeans to account for South Asians' greater stroke risk.^30^ Both our study and national surveys report ethnic differences in BP that are nothing like this, however. Furthermore, other key contributors, such as smoking and total cholesterol, are favorable in South Asians. Diabetes prevalence maps more closely to the ethnic subgroup gradient in stroke, being highest in Bangladeshis, intermediate in Pakistanis and Indians, and lowest in Europeans.^5^

South Asians have higher HbA_1c_ levels than do Europeans, and statistical adjustment for HbA_1c_ attenuated the ethnic differences in hemodynamic recovery, an effect that was similar to the effect of resting BP. However, the effect of HbA_1c_ was overwhelmed in the multivariable model by baroreceptor function and PWV, which suggests that the effect of hyperglycemia on recovery is not direct but is mediated via autonomic function and aortic stiffness. The persistently elevated BP in recovery in South Asians, related to their greater hyperglycemia, might provide a potential explanation for the mapping of stroke mortality rates to diabetes mellitus prevalence gradients in South Asian subgroups. A recent review supports this assertion^32^: Lacunar infarcts are more commonly observed in South Asians, and these are thought to be more strongly associated with the microvascular disease of hyperglycemia.

Mechanisms linking hyperglycemia to alterations in hemodynamic responses to exercise, and thus downstream cardiovascular risk, are uncertain. We have reported previously that insulin resistance and hyperglycemia are associated with impaired sympathovagal balance in South Asians.^26^ Normally, enhanced vagal tone after exercise drives recovery of BP and RPP^33^; attenuated recovery is due to impairment of this response.^34^ Aortic stiffness also has been proposed as a mechanism to account for abnormal recovery BP and RPP.^35^ Arterial stiffness is increased in individuals with impaired endothelial function, as has been reported in South Asians,^36^ and also contributes to impairment of the baroreflex.^37^ Interestingly, aortic stiffness is elevated in South Asians after stroke^38^ because of their greater hyperglycemia. We show that elevated aortic stiffness in South Asians contributes to attenuated BP and RPP postexercise recovery, and that this association adds to and is independent of baroreflex sensitivity. We propose, therefore, that hyperglycemia in South Asians results in poor autonomic function and greater arterial stiffness and that these disturbances in turn adversely affect hemodynamic responses to exercise.

This study has limitations. Causal relationships cannot be established from cross-sectional analyses; however, it seems plausible that adverse autonomic function would predict BP and RPP responses to exercise, rather than the converse. The majority of previous studies have used a treadmill test or cycle ergometer as the exercise stressor. However, the utility of exercise BP responses in predicting cardiovascular outcomes does not seem to be dependent on the stressor,^5^ and the modest degree of exercise used in the present study is more likely to reflect real-life exposures. Antihypertensive medication could influence the hemodynamic response to exercise. We did not exclude individuals on medication from the study because this would have resulted in an unrepresentative biased sample, but analysis restricted to those not receiving antihypertensive medication was similar to that for the whole sample. Furthermore, adjustment for medication use did not account for the ethnic differences in hemodynamic response. People with established CVD were excluded, and because participants already had undergone detailed subclinical investigation, it is unlikely that people with predisposing disease (eg, carotid stenosis) would have been missed. This study was restricted to South Asians of Punjabi Sikh origin. All South Asian subgroups share an elevated risk of stroke, all have largely similar or lower resting BP compared to Europeans, and all have a greater prevalence of hyperglycemia and insulin resistance. We therefore suggest that it is plausible that the mechanisms described here might apply more widely to all South Asian subgroups, although this would require testing in each group to be definitive. The hyperglycemia and insulin resistance could be common precursors of both autonomic dysfunction and increased arterial stiffness.^39^ Genetic explanations have been invoked to account for ethnic differences in disease, yet the search for genes determining stroke risk has proved disappointing, with little evidence for ethnic differences.^40^ There is clearly scope for residual confounding, either because of the impact of unmeasured factors or because of imprecise assessment of our key risk factors, as a single BP or HbA_1c_ measurement will not completely reflect long-term BP or glycemic status. Finally, although our proposed explanation of the mechanisms underlying adverse exercise-related hemodynamic responses is plausible, we acknowledge that we have not comprehensively tested all potential mechanisms, such as downstream effects of hyperglycemia on endothelial function.

BP is the strongest contributor to cardiovascular risk. We demonstrate the importance of detailed phenotypic characterization, which includes dynamic as well as static measures, to further understand population differences in disease risk and their underlying mechanisms. These tests also might prove to be of value in future clinical decision making. Impaired BP recovery after exercise is a potential mechanism explaining the increased risk of stroke and CVD in South Asians, but clearly this needs to be tested in prospective studies. Blood glucose lowering can improve autonomic function in hyperglycemic states,^41^ and exercise training significantly improves hemodynamic response to exercise,^42^ thus providing potential routes by which cardiovascular risk could be reduced in South Asians.
